# ICF Priorities and the Impact of Demographic Factors in a Paediatric Poststroke Population: An Observational Study

**DOI:** 10.1111/cch.70352

**Published:** 2026-09-23

**Authors:** Leanne Dreyer, Ibidun Fakoya, Capucine Jauffret, Naomi de Souza‐Figueiredo

**Affiliations:** ^1^ Paediatric Neurosciences, Evelina London Children's Hospital Guy's and St Thomas' NHS Foundation Trust London UK; ^2^ Department of Women and Children's Health, School of Life Course and Population Sciences Kings College London London UK; ^3^ Kings Health Partners Women and Children's Health Clinical Academic Partnership London UK; ^4^ Department of Psychology University of Bath Bath UK

**Keywords:** child, equity, neurological, paediatric, participation, rehabilitation, stroke

## Abstract

**Background:**

Common outcomes of childhood stroke include physical, cognitive, psychological and social difficulties. Evidence indicates young people with acquired brain injury have a range of community neuropsychological priorities impacting participation; however, less is known about priorities following childhood stroke specifically, and the demographic influences on these. This knowledge is essential to ensure rehabilitation services meet their needs.

**Methods:**

Two‐hundred twenty‐two clinical priorities were extracted from the medical records of 59 children (66% male, 59% White, aged 1–17 years, median 7, all deprivation quintiles) attending an occupational therapy and neuropsychology Stroke Recovery Clinic: stroke type: 51% perinatal and 49% childhood.

Priorities were coded using the International Classification of Functioning Disability and Health (ICF). Chi‐square analysis identified associations between priorities and demographic factors.

**Results:**

On average, four priorities were raised per patient. Priorities were distributed across ICF components, with 47% classified as activities and participation, 25% as body functions, 23% as environmental factors and 5% as body structures. Key concerns included behaviour/emotion, health/education navigation, fine hand use and societal attitudes. No significant demographic differences were found. A nonsignificant trend showed more body function and environmental priorities in primary and high school. Males and non‐White patients continued clinic access into adolescence, with fewer non‐White individuals in preschool.

**Discussion:**

Children and their families have a range of priorities post‐stroke, requiring patient and family centred, interdisciplinary and context specific approaches. These findings support longitudinal, integrated hospital, community and school care systems, which focus on children and young people's participation in their homes and communities well beyond the time of their stroke. Services must ensure interdisciplinary post‐stroke care for children and young people is equitable and accessible, with priorities addressed in a timely manner.

## Summary

- Services for children and young people post‐stroke should be designed around patient and family needs, which prioritise children and young people's participation in their homes and communities.
- An interdisciplinary approach allows for integrated and holistic care for children and young people post‐stroke, with coordination across hospital, community and education systems.
- Access to evidence‐based rehabilitation poststroke needs to be equitable and accessible.
- Services should provide longitudinal support in order to capture the needs, which arise at different timepoints.

## 1 Introduction

Childhood stroke, occurring from the perinatal period up to age 18, affects approximately 1.2–13 per 100 000 children and is the leading cause of acquired brain injury in this age group (Rawanduzy et al. 2022). In fact, these estimates may be low due to clinical underrecognition and potential misdiagnosis, particularly in communities with limited healthcare access (Rawanduzy et al. 2022). Stroke in childhood impacts brain development, with outcomes varying based on factors such as diagnosis, timely access to acute treatment, rehabilitation resources and socioeconomic background (Jordan et al. 2018). Notably, social determinants such as low income are associated with poorer neurological outcomes, demonstrating the importance of addressing health inequalities in paediatric stroke care (Jordan et al. 2018). Common long‐term effects of childhood stroke include physical, cognitive, psychological and social challenges, often requiring comprehensive, sustained support across the life span (Mackay and Steinlin 2019). The impact of functional difficulties can vary by developmental stage, as children and families encounter evolving challenges throughout the different life stages.

The World Health Organization's International Classification of Functioning, Disability and Health (ICF) provides a comprehensive bio‐psychosocial framework that can be used to evaluate the impact of disability, including childhood stroke, across multiple domains (World Health Organization 2001). These domains include body structures, body functions, activities and participation, and environmental factors. It complements the eleventh edition of the International Classification of Diseases, which describes disease, disorder and injury, by describing the functional impact of a diagnosed condition (World Health Organization 2022).

The ICF has been instrumental in advancing understanding of community neurorehabilitation goals in children with acquired brain injury (McCarron et al. 2019). In a previous study with this population, most goals were classified within activities and participation (52%), emphasising the importance of supporting young people's engagement in everyday activities and social participation. Additional needs related to body functions (28%), environmental factors (20%) and body structures (< 1%) were also identified needs (McCarron et al. 2019).

Literature and the National Institute for Health and Care Excellence accredited *Stroke in Childhood—clinical guideline for diagnosis, management and rehabilitation* (Royal College of Paediatrics and Child Health SA 2017), indicate that post‐stroke services for children and young people require an individualised, multidisciplinary, goal‐based and context specific approach, with a strong focus on activities and participation (Gordon et al. 2018; McCarron et al. 2019). However, children and families experience high levels of unmet needs across health domains following paediatric stroke (Gordon et al. 2018). Some children experience delays in receiving a diagnosis and in accessing acute treatment and evidence‐based rehabilitation. In the United Kingdom, variations in rehabilitation services nationally also contribute to disparities in post‐stroke care for children and adolescents (Hayes et al. 2017). In addition, systematic specification of paediatric rehabilitation services remains limited in comparison to adult services (Hayes et al. 2017).

Research in other paediatric neurodevelopmental populations has found differences according to sex and ethnicity as to how services are provided (Slobodin and Masalha 2020; Martin et al. 2024). A review of therapy services within Southeast England for children and families with complex needs has described some economic barriers that impact access to and delivery of services. As a result, some children are impacted by inconsistent or inadequate services (Randall et al. 2024).

Therefore, it is essential to capture the needs and voices of children, young people and their families to shape responsive rehabilitation services post‐stroke. Given the diversity in paediatric post‐stroke populations, understanding how priorities vary by age, time post‐injury, sex, ethnicity and social background is essential. Exploring these demographic factors can hopefully lead to a more equitable approach to rehabilitation that addresses health inequalities and supports the developmental needs of children, young people and their families.

### 1.1 Aim

The aim of this study is to explore the relationships between priorities and demographic factors of children, young people and their families post‐stroke.

## 2 Methods

### 2.1 Setting

This study was conducted within a specialist, tertiary, outpatient service for children and young people following a stroke or acquired brain injury, delivered within the context of the UK National Health Service (NHS). The occupational therapist and neuropsychologist‐led interdisciplinary Stroke Recovery Clinic provides developmental surveillance, consultation and rehabilitation advice for children, young people (0–18 years) and their families.

Priorities are identified by the children, young people and/or their families, at the beginning of each clinic appointment, with clinicians asking open‐ended questions to elicit these. These priorities inform the focus of advice and information provided in clinic appointments. Evidence‐based assessment and rehabilitation recommendations in relation to these priorities are recorded in clinic summary letters. Context‐specific, child‐centred principles underpin this service to ensure service users' priorities are considered holistically in partnership with educational settings and local therapy teams.

The service aims to offer appointments when clinically indicated at a minimum of once every 12 months. The children, young people and their families are supported to integrate into local, community‐based services, at which point they are discharged from the tertiary service, with re‐referral as required.

### 2.2 Patients

Patients were identified from a database of service users who attended the specialist service during an 18‐month period between September 2021 and March 2023. All those who attended the service during that time and who met inclusion criteria were included in the analysis.

Referral criteria for the service include children and young people aged 0 months–18 years who have experienced a stroke perinatally or during childhood and who have function and participation needs. Referrals originate from primary care, hospital‐based or community‐based medical professionals within the London and Southeast region. Referred children are not required to have a named neurologist.

Where patients had multiple visits, only their most recent visit was included. This yielded 61 children and young people. Fifty‐nine children and young people who had experienced a stroke were included in this analysis. Patients were excluded if they had experienced a brain injury other than a stroke.

### 2.3 Data Extraction

Child, young person and family priorities, age at clinic, age at stroke, sex, ethnicity, postcode and time since stroke were extracted from clinic records. Patients were grouped into age categories for age at clinic: preschool (0–4 years), primary school (5–10 years) and high school (11–18 years). The English Indices of Multiple Deprivation (IMD) 2019 (Noble et al. 2019) were used to measure relative deprivation of patients at a local area level. The NHS data dictionary was used to describe patient ethnic identity (NHS 2021).

### 2.4 Coding

Priorities were independently coded using the ICF by 3 researchers (L.D., C.J. and N.d.S.F.) following its guidelines for coding and descriptions within the ICF (World Health Organization 2001) and in line with previous research in this area (McCarron et al. 2019). The ICF features four components: body structures, body functions, activities and participation, and environmental factors. The ICF provides a framework to quantitively explore the impact health conditions have on a child's functioning in daily life and allow for comparison with previous research. Diagnostic labels (e.g., autism) are not used within this framework, and instead, the functional impacts are described.

The ICF (World Health Organization 2001) follows an alphanumeric coding system. Each component links to a letter: body structure = ‘s’, body function = ‘b’, activities and participation = ‘d’ and environmental factors = ‘e’. The first number that follows the letter denotes the chapter and the two digits after that link to the branching levels.

Initially concordance between two and three authors was seen for 73.4% of codes (52.7% and 20.7%). Discussion occurred until concordance was reached at the ICF second‐level classification code. Where possible, a single ICF code was sought to represent each rehabilitation priority. If necessary, priorities relating to different aspects of the ICF were split and allocated different codes to accurately represent each need in accordance with the ICF guidelines.

### 2.5 Statistical Analysis

All data were double entered onto Microsoft Excel 365 (Microsoft t Corp) and analysed using Stata statistical software Version 18.0 (StataCorp LP). Significance testing was set at two‐sided *p* < 0.05.

The STROBE Checklist was used to ensure transparent and robust reporting (von Elm et al. 2007).

## 3 Results

### 3.1 Patient Demographics

Patients were 66% male, 59% White, aged 1–17 years at appointment (median = 7 years; 22% preschool, 58% primary school and 20% high school). All deprivation quintiles were represented. 51% of patients had perinatal stroke, 49% childhood stroke (of which 45% occurred < 1). Time since stroke was between 0 and 17 years (median = 5 years).

Patient attendance during the study period suggests that poststroke needs evolve over time, with most clinic attendees being of primary school age. Within the clinic, boys were seen twice as often as girls. Notably, males and non‐White individuals continued to access the specialist tertiary multidisciplinary clinic into adolescence, whereas a lower proportion of non‐White individuals accessed the clinic during preschool years (Tables 1 and 2).

**TABLE 1:** Sociodemographic characteristics by gender.

	Gender					
	Male		Female		Total	
	*N*	(%)	*N*	(%)	*N*	(%)
Age group						
Preschool	11	(28.2)	2	(10.0)	13	(22.0)
Primary school	18	(46.2)	16	(80.0)	34	(57.6)
High school	10	(25.6)	2	(10.0)	12	(20.3)
Ethnicity						
White	24	(61.5)	11	(55.0)	35	(59.3)
Non‐White	15	(38.5)	9	(45.0)	24	(40.7)
Index of multiple deprivation (quintiles)						
1 (most deprived)	4	(10.3)	5	(26.3)	9	(15.5)
2	10	(25.6)	2	(10.5)	12	(20.7)
3	12	(30.8)	4	(21.1)	16	(27.6)
4	8	(20.5)	2	(10.5)	10	(17.2)
5 (least deprived)	5	(12.8)	6	(31.6)	11	(19.0)
Mean number of priorities (SD)	3.7	(1.8)	3.9	(1.2)	3.8	(1.6)
Body functions	0.7	(0.5)	0.6	(0.5)	0.6	(0.5)
Activities and participation	0.8	(0.4)	0.9	(0.3)	0.8	(0.4)
Environmental	0.5	(0.5)	0.7	(0.5)	0.6	(0.5)
Body structures	0.2	(0.4)	0.2	(0.4)	0.2	(0.4)

**TABLE 2:** Sociodemographic characteristics by ethnicity.

	Ethnicity					
	White		Non‐White		Total	
	*N*	%	*N*	%	*N*	%
Age group						
Preschool	11	(31.4)	2	(8.3)	13	(22.0)
Primary school	21	(60.0)	13	(54.2)	34	(57.6)
High school	3	(8.6)	9	(37.5)	12	(20.3)
Index of multiple deprivation (quintiles)						
1 (most deprived)	5	(14.7)	4	(16.7)	9	(15.5)
2	6	(17.6)	6	(25.0)	12	(20.7)
3	7	(20.6)	9	(37.5)	16	(27.6)
4	7	(20.6)	3	(12.5)	10	(17.2)
5 (least deprived)	9	(26.5)	2	(8.3)	11	(19.0)
Mean number of priorities (SD)	3.7	(1.2)	3.9	(2.1)	3.8	(1.6)
Body functions	0.7	(0.4)	0.5	(0.5)	0.6	(0.5)
Activities and participation	0.9	(0.4)	0.8	(0.4)	0.8	(0.4)
Environmental	0.5	(0.5)	0.6	(0.5)	0.6	(0.5)
Body structures	0.1	(0.3)	0.3	(0.4)	0.2	(0.4)

### 3.2 Patient and Family Priorities Across ICF Components and Chapters

A total of 222 priorities were identified. On average, 3.76 priorities were raised per patient, with no significant differences seen according to gender and ethnicity (Tables 1 and 2). Of priorities raised, those in the activities and participation component were the most reported (47%). Twenty‐five percent were body function, 23% environmental factors and 5% body structures (Table 3). Table 4 outlines the most frequently reported patient and family priorities, which are spread across all 4 ICF components.

**TABLE 3:** Distribution of patient and family priorities across ICF components and chapters.

Components and chapters	ICF chapter code	N of codes present	Codes present based on total sample (%)
Activities and participation		105	(47.3%)
1. Learning and applying knowledge	d1	7	(3.2%)
2. General tasks and demands	d2	21	(9.5%)
3. Communication	d3	7	(3.2%)
4. Mobility	d4	19	(8.6%)
5. Self‐care	d5	31	(14.0%)
7. Interpersonal interactions and relationships	d7	9	(4.1%)
8. Major life areas	d8	6	(2.7%)
9. Community social and civic life	d9	5	(2.3%)
Body functions		55	(24.8%)
1. Mental functions	b1	38	(17.1%)
2. Sensory functions and pain	b2	4	(1.8%)
3. Voice and speech functions	b3	1	(0.5%)
5. Functions of the digestive, metabolic and endocrine systems	b5	2	(0.9%)
7. Neuromusculoskeletal and movement related functions	b7	10	(4.5%)
Environmental factors		52	(23.4%)
3. Support and relationships	e3	1	(0.5%)
4. Attitudes	e4	18	(8.1%)
5. Services, systems and policies	e5	33	(14.9%)
Body structures		10	(4.5%)
1. Structure of the nervous system	s1	7	(3.2%)
4. Structures of the cardiovascular, immunological and respiratory systems	s4	1	(0.5%)
7. Structures related to movement	s7	2	(0.9%)

*Note:* The ICF (Royal College of Paediatrics and Child Health SA 2017) follows an alphanumeric coding system. Each component links to a letter: body structure = ‘s’, body function = ‘b’, activities and participation = ‘d’ and environmental factors = ‘e’. The first number that follows the letter denotes the subchapter (first level) and the two digits after that link to the branching level (second level).

**TABLE 4:** Most frequently reported priorities.

Branching level	ICF code	*N* of codes present	Codes present based on total sample (%)
Handling stress and other psychological demands	d240	18	8.1%
Health services, systems and policies	e580	18	8.1%
Emotional functions	b152	17	7.7%
Fine hand use	d440	15	6.8%
Education and training services, systems and policies	e585	14	6.3%
Societal attitudes	e460	12	5.4%
Sleep functions	b134	11	5.0%
Dressing	d540	9	4.0%
Eating	d550	7	3.2%
Toileting	d530	7	3.2%
Structure of brain	s110	7	3.2%

### 3.3 Activities and Participation

The majority of the priorities came under the Activities and Participation component. The joint most frequently reported priority was handling stress and other psychological demands (8.1% of all reported priorities). Examples of this were ‘started to have meltdowns’, and ‘struggles with transitions between activities’, and ‘doesn't always listen’. Fine hand use was the fourth most frequently reported priority (6.8%; e.g., ‘interaction with toys using both hands’). The functional impact on dressing (4%), eating (3.2%) and toileting (3.2%) was also frequently discussed, noting the impact of both physical and cognitive factors, for example, ‘pulling up pants after the toilet’, ‘things [functions] become more difficult under stress’ and ‘dealing with hunger and requesting food’.

### 3.4 Body Functions

Difficulties with emotional functions were third most frequently reported (7.7%) and included ‘frustration and sadness when she can't do something’ and ‘feeling strange, panicky and quiet’. Sleep was the seventh most reported priority (5%), which was often related to behavioural issues, for example, ‘takes a long time to settle’ and ‘sleep and fatigue’.

### 3.5 Environmental Factors

Health Services, Systems and Policies (8.1%) was joint most frequently reported as a priority, and Education and Training Services, Systems and Policies (6.3%) was fifth. Examples raised were ‘clarity and more involvement from occupational therapy and physiotherapy’, ‘concerned about lack of support at school’ and ‘advice and guidance to access support to manage GCSE examinations’. Also, frequently reported under this component were Societal Attitudes (5.4%), which included comments about children being ‘self‐conscious and isolated from others’. There were also questions about ‘how to have a conversation with him about his medical history, in the context of his growing awareness of differences’.

### 3.6 Body Structures

The only factor in this component that was frequently reported was ‘Structure of the brain’ (3.2%), which was used to code neurological issues such as seizures or headache.

### 3.7 The Relationship Between Demographics and Priorities

Chi‐square analysis indicated no significant difference in the relative proportion of priorities across components according to sex (*p* = 0.92), ethnicity (*p* = 0.44) and deprivation (*p* = 0.84), with the majority of priorities categorised into Activities and Participation for all groups (data not shown). There was a nonsignificant trend towards increased reporting of body function and environmental priorities in primary and high school (*p* = 0.25).

**FIGURE 1:**
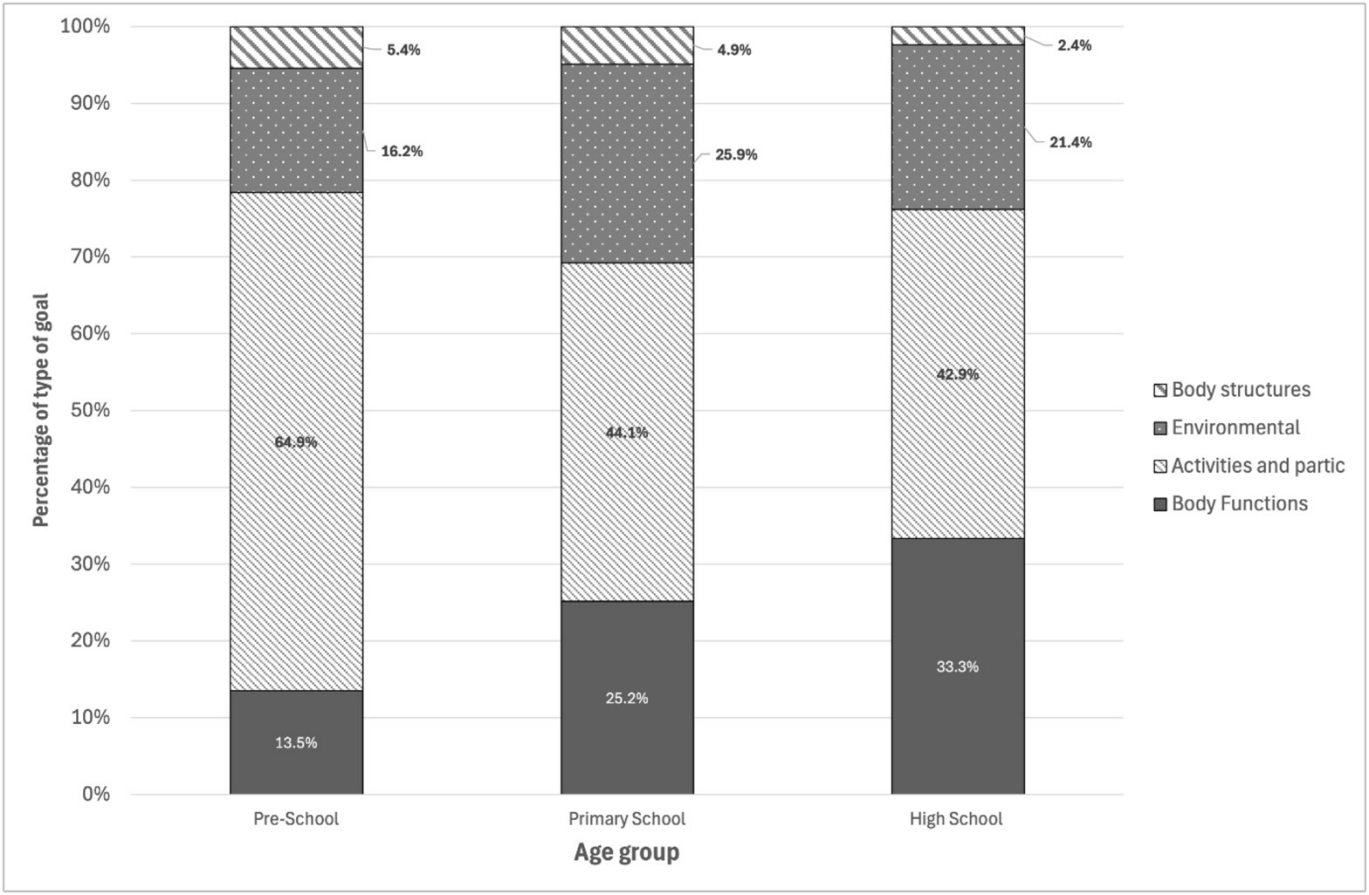
Relative proportion of priorities across ICF components according to school stage.

Of all the priorities raised by preschoolers, 13.5% were about body function, compared with 25.2% among primary schoolers and 33.3% among those in high school (Figure 1). Compared to 38.5% of preschoolers who raised body function priorities compared with 70.6% of primary schoolers and 75% of high schoolers.

## 4 Discussion

The aim of this study was to improve the understanding of the priorities of children, young people and their families who attended a tertiary interdisciplinary stroke recovery outpatient clinic.

The nature and breadth of identified priorities are consistent with previous research in children with acquired brain injury (Gordon et al. 2018; McCarron et al. 2019) with activities and participation components being the most reported. This highlights that an interdisciplinary clinic model, where the most frequently reported aspects of activities and participation (e.g., self‐care, general tasks and demands and mobility) are supported, can be an effective way of meeting the needs of children young people and families. The model must have the flexibility and opportunities for clinicians to develop the skills and knowledge to assess and formulate the influence of physical, cognitive, emotional and social factors on the reported challenges for children and young people longitudinally across development. This is in line with the NHS England requirements of Paediatric Neurorehabilitation and Paediatric Neurodisability (NHS England 2013), which outlines the need for support for emotional health and wellbeing of children, specialist interdisciplinary assessment and ongoing support, as well as coordination of care between health, community therapy and education services.

The high proportion of priorities raised in the environmental factors component suggests that patient needs are not being met through current service structures. Systems, services and policies priorities were frequently raised relating to education, and to health and housing, where services were inadequate or not in place. This is in line with previous analysis of rehabilitation services in the United Kingdom (Hayes et al. 2017) and likely reflects a lack of awareness at government and policy levels, affecting fund allocation (Jim et al. 2022).

For school aged children, school is often where recovery and rehabilitation can take place, and there are education policies and processes in place to support children with additional needs. However, teachers and childcare professionals have been found to have limited awareness and knowledge regarding the needs of poststroke patients (Bennett et al. 2022) so rehabilitation opportunities may be missed. This study demonstrates some differences in reported priorities according to demographic factors. As seen in previous research (McCarron et al. 2019), there is a trend towards an increase in Environmental priorities being raised as children enter primary and secondary school. This demonstrates the importance of a flexible clinic model that allows for greater emphasis on access and participation needs as children approach adolescence.

A second frequently reported priority in the environmental component was attitudes, including broad societal attitudes, as well as specific attitudes of family members and professionals. Despite disability being a protected characteristic under the 2010 Equalities Act (GOV.UK 2010), children and families reported instances of discriminatory behaviour impacting on participation. In addition, there were frequent reports of children and young people struggling with a feeling of ‘difference’ relating to their impairments post‐stroke, reflecting the impact of societal attitudes and the absence of a widely held social model of disability (Haegele and Hodge 2016). Community acceptance and support have been found to impact participation for youth with acquired brain injury (de Kloet et al. 2015), so it is vital that these broader issues are addressed to reduce environmental barriers to access. For example, research has demonstrated positive impact of psychoeducational interventions about acquired brain injury on stigmatisation in non‐injured young people (Irwin and Fortune 2014).

The study captured a clinical population routinely attending an outpatient clinic, with patients representing a range of demographic backgrounds (sex, ethnicity, school stage and deprivation index). Males and non‐White individuals continued to access the specialist tertiary multidisciplinary clinic into adolescence, and non‐White individuals were less represented at preschool. This suggests that tertiary and community services are not identifying and meeting the needs of individuals equally. This aligns with broader findings in neurodevelopmental populations (Martin et al. 2024; Randall et al. 2024).

However, unlike previous research, differences in outcomes based on deprivation level were not observed (Jordan et al. 2018). Wider literature notes unequal exposure and access to care at the first point of contact post‐stroke in adult populations; however, once the care pathway has been established, as is the case when accessing this tertiary service, some socioeconomic barriers may not be as impactful (Bray et al. 2018). Studies tracking healthcare delivery metrics over time demonstrate that a universal framework structurally narrows equity gaps in healthcare process quality and general practice supply over time (Cookson et al. 2016).

The absence of a national paediatric stroke database and clinical guidelines creates a challenge for clinicians and policymakers in this area, as compared to the adult stroke population (Hayes et al. 2017). It is therefore vital that clinicians working with this population collaboratively and consistently gather clinical data for quality control and sharing of expertise. Capturing the voices of service users is also key to ensuring services meet the needs and priorities of children, young people and their families post stroke. Stroke policy, clinical guidelines and research within the United Kingdom focus primarily on adults, even though neonatal stroke risk is among the highest across the lifespan (Srivastava and Kirton 2021).

The James Lind Alliance research priorities for Childhood Neurological Conditions include research into sleep, emotional wellbeing, nonmedicinal intervention for motor‐disorders, feeding and rehabilitation (Alliance: JL 2022). It is notable that these were all issues raised in this clinic, highlighting the importance of these areas to the paediatric poststroke population. Research into interventions and service models for these areas would enable more specific and evidence‐based referrals and recommendations to be made. The ICF is a valuable tool for mapping priorities for children and families post‐stroke in research and has been used in developing a comprehensive core set for children and young people with Cerebral Palsy to describe function as well as barriers and facilitators which influence function specifically for children and young people with Cerebral Palsy (Schiariti et al. 2015). At a service level, it can be used as a framework for goal setting that incorporates functional, participation and environmental goals (Schiariti et al. 2015; Leonardi and Fheodoroff 2021; Boakye and Mwale 2022).

### 4.1 Limitations

This study extends previous research by focussing specifically on paediatric stroke. However, the majority of patients accessing the clinic (73%) had experienced perinatal or childhood stroke prior to the age of 1, which limits the generalisability of the findings. Analysis of results according to the age at which stroke occurred and the time since stroke was not included, due to the limited range in this data. Although some differences were seen in needs based on the educational/developmental stage of a child at clinic, it was not possible to explore how this interacts with the more acute medical stroke recovery process, or the family/child adjustment to post‐stroke needs.

Furthermore, clinical information about the nature of the stroke was not included in the analysis due to insufficient information available from clinical records (e.g., some strokes were clinically diagnosed without scans). The ‘recovery continuum’ model suggests that neural and behavioural recovery processes are influenced by time of injury and interact with developmental, environmental and brain structural influences (Anderson et al. 2011). This study therefore does not capture the complexity of factors that may influence priorities, limited by the ‘real‐world’ challenges of clinical audit with incomplete data and records.

Children and young people have a range of priorities post‐stroke requiring patient and family centred, interdisciplinary and context specific approaches. These findings support longitudinal, integrated hospital, community and school care systems, which prioritise children and young people's participation in their homes and communities, well beyond the time of their stroke. Services must ensure that interdisciplinary post‐stroke care for children and young people is equitable and accessible, with child, young people and family priorities addressed in a timely manner.

## Author Contributions

**Leanne Dreyer:** writing – original draft, writing – review and editing, conceptualization, investigation, methodology, validation, formal analysis, project administration, data curation. **Ibidun Fakoya:** formal analysis, writing – original draft, writing – review and editing, software, data curation. **Capucine Jauffret:** conceptualization, investigation, writing – review and editing, formal analysis, data curation, project administration, methodology, validation. **Naomi de Souza‐Figueiredo:** conceptualization, investigation, writing – original draft, methodology, validation, writing – review and editing, formal analysis, project administration, data curation.

## Funding

The authors have nothing to report.

## Ethics Statement

NHS internal approval for the use of deidentified routinely collected clinical data was provided by Guy's and St Thomas' NHS Foundation Trust; service evaluation number 14560. All data were obtained as part of routine clinical care and stored and analysed in compliance with data protection legislation and local National Health Service Trust regulations.

## Conflicts of Interest

L.D. and N.d.S.F. hold clinical management roles overseeing the Stroke and Acquired Brain Injury Recovery Service from which the data were collected. The authors declare there are no further conflicts of interest and that the views expressed are our own and not necessarily those of the NHS.

## Data Availability

Research data are not shared.
